# Family supplemented patient monitoring after surgery (SMARTER): a pilot stepped-wedge cluster-randomised trial

**DOI:** 10.1016/j.bja.2024.06.027

**Published:** 2024-07-27

**Authors:** Adam Hewitt-Smith, Fred Bulamba, Akshaykumar Patel, Juliana Nanimambi, Lucy R. Adong, Bernard Emacu, Mary Kabaleta, Justine Khanyalano, Ayub H. Maiga, Charles Mugume, Joanitah Nakibuule, Loretta Nandyose, Martin Sejja, Winfred Weere, Timothy Stephens, Rupert M. Pearse

**Affiliations:** 1 Faculty of Medicine and Dentistry, Queen Mary University of London, London, UK; 2 Department of Anaesthesia and Critical Care, Faculty of Health Sciences, Busitema University, Mbale, Uganda; 3 Elgon Centre for Health Research and Innovation (ELCHRI), Mbale, Uganda; 4 Comprehensive Rehabilitation Services in Uganda (CoRSU) Hospital, Kisubi, Uganda; 5 Nexus Centre for Research and Innovations (NCRI), Wakiso, Uganda

**Keywords:** Africa, failure to rescue, low- or middle-income country, low-resource setting, postoperative care, surgery, task sharing

## Abstract

**Background:**

Mortality after surgery in Africa is twice that in high-income countries. Most deaths occur on wards after patients develop postoperative complications. Family members might contribute meaningfully and safely to early recognition of deteriorating patients.

**Methods:**

This was a stepped-wedge cluster-randomised trial of an intervention training family members to support nursing staff to take and record patient vital signs every 4 h after surgery. Adult inpatients across four surgical wards (clusters) in a Ugandan hospital were included. Clusters crossed once from routine care to the SMARTER intervention at monthly intervals. The primary outcome was frequency of vital sign measurements from arrival on the postoperative ward to the end of the third postoperative day (3 days).

**Results:**

We enrolled 1395 patients between April and October 2021. Mean age was 28.2 (range 5–89) yr; 85.7% were female. The most common surgical procedure was Caesarean delivery (74.8%). Median (interquartile range) number of sets of vital signs increased from 0 (0–1) in control wards to 3 (1–8) in intervention wards (incident rate ratio 12.4, 95% confidence interval [CI] 8.8–17.5, *P*<0.001). Mortality was 6/718 (0.84%) patients in the usual care group *vs* 12/677 (1.77%) in the intervention group (odds ratio 1.32, 95% CI 0.1–14.7, *P*=0.821). There was no difference in length of hospital stay between groups (usual care: 2 [2–3] days *vs* intervention: 2 [2–4] days; hazard ratio 1.11, 95% CI 0.84–1.47, *P*=0.44).

**Conclusions:**

Family member supplemented vital signs monitoring substantially increased the frequency of vital signs after surgery. Care interventions involving family members have the potential to positively impact patient care.

**Clinical trial registration:**

NCT04341558.

**Editor's key points**

• Mortality after surgery in Africa is twice that in high-income countries, with most deaths occurring on patient wards.
• The potential of family members to contribute to early recognition of deteriorating patients was assessed in a pilot stepped-wedge cluster-randomised trial.
• The intervention involved training family members to support nursing staff to take and record patient vital signs every 4 h after surgery.
• In a study of 1395 patients, family member supplemented vital signs monitoring increased the frequency of vital signs after surgery.
• The ability of care interventions involving family members to impact patient outcomes requires further study in effectiveness trials.

The African Surgical Outcomes Study (ASOS) provided, for the first time, robust surgical outcomes data from 25 nations across the continent. Compared with global averages, the typical surgical patient in Africa is twice as likely to die, despite being younger and with fewer comorbidities.^1^ For some surgical procedures, such as Caesarean delivery, mortality rates in Africa are 50 times higher than those in high-income countries,^2^ and 94% of deaths occur on the hospital wards after surgery.^1^ Other outcomes studies across Africa have also demonstrated that the majority of deaths occurring after surgery happen during the postoperative period.^3^^,^^4^ These deaths represent a ‘failure to rescue’: death of a patient following unrecognised physiological deterioration caused by a complication of surgery or anaesthesia.^5^^,^^6^

As surgical complication rates are similar across low-, middle-, and high-income settings, processes that lead to ‘failure to rescue’ are likely to be responsible for the higher mortality rates observed across Africa. The capacity of health facilities to rescue deteriorating patients is, in part, dependent on health system factors, for example access to critical care or advanced radiology services.^7^ However, failure to appropriately monitor a patient, to recognise physiological deterioration, and to act when deterioration occurs also contribute to ‘failure to rescue’.^8^ Workforce shortages across Africa can result in patient-to-nurse ratios of up to 60:1 making close postoperative monitoring difficult to achieve.^9^ In Ugandan hospitals, patient family members provide much of the personal care that nurses deliver in high-income countries.^10^ In many cases, family members sleep beside or under the patient's hospital bed throughout their in-patient stay. Outside of surgical care, family members have been shown to contribute safely and positively to other routine aspects of patient care.^11^, ^12^, ^13^

We hypothesised that family carers could be taught to conduct basic vital signs monitoring, to supplement the monitoring of patients by nurses on postoperative wards. If successful, this would contribute to improved postoperative surveillance, allowing early intervention when complications occur and the prevention of deaths after surgery. In the SMARTER pilot trial, we tested the efficacy of an intervention to train family members to perform basic vital signs monitoring to supplement routine postoperative monitoring by staff in a regional hospital in Uganda.

## Methods

The trial was registered prospectively at ClinicalTrials.gov (NCT04341558). Ethics approval was granted by Mbale Regional Referral Hospital (RRH) Research Ethics Committee (MRRH-2020-7), Uganda National Council for Science and Technology (HS944ES), and the Queen Mary Ethics of Research Committee (QMERC2019/72).

### Setting

The trial took place at Mbale RRH for 6 months in 2021. Mbale RRH is a 470-bed teaching hospital in eastern Uganda serving a population of 4.5 million people. Four independent inpatient wards in the hospital provide care for patients after they have undergone surgery. Two wards providing postoperative care are dedicated surgical wards, and two are mixed obstetric/gynaecology wards; each ward contains 20–40 beds. Patient-to-nurse ratios on the inpatient surgical wards routinely reach 40:1. Surgical and maternity high-dependency units provide care for the sickest patients. At the time of this trial, the hospital did not have an ICU and could not provide organ support (e.g. mechanical ventilation or renal dialysis). Medical and nursing teams provide care for patients on their wards only, and movement of patients between wards is rare.

### Study design

SMARTER was a stepped-wedge cluster-randomised pilot trial. Each inpatient surgical ward in the hospital represented a cluster. The order in which the trial intervention was implemented through the clusters was determined by computer-generated randomisation performed before the start of the trial by an independent statistician at the Pragmatic Clinical Trials Unit at Queen Mary University of London. Only the lead investigator in Uganda knew the order of randomisation in advance. The study team and ward staff were informed 2 days before their transition to enable preparation. Each cluster had usual care and intervention phases with the intervention being introduced into one cluster each month from month 2 to month 5 (Supplementary Fig. S1). Each cluster transitioned once from usual care to intervention at 08:00 on the first day of that month period. If the patient's date of surgery fell after this time, they were recruited into the intervention arm. A concurrent process evaluation of the intervention was undertaken by the study team, which will be reported separately.^14^

### Participants

All patients who underwent surgery and were admitted to one of the four postoperative surgical wards during the trial period were screened for eligibility. Patients or a carer needed to speak one of five commonly used languages. Written informed consent was provided by patients during the usual care phase and by both the patient and their family member acting as their main carer during the intervention phase. Additional inclusion criteria during the intervention phase included the following: the carer's ability to know the time of day, using either a mobile phone or a wall clock, the ability to read numbers from the pulse oximeter, and the ability to document on an observation chart that the vital signs had been taken. Exclusion criteria included the following: patients <5 yr old, patients discharged on the same day of surgery, and patients who were identified 24 h or more after their operation.

### Trial intervention

The trial intervention of training and supporting family carers to take basic vital signs for their patient family member after surgery was developed following Medical Research Council/National Institute for Health Research guidance.^15^ We used an iterative intervention development process with stakeholder engagement from medical and nursing teams in the hospital and feedback from family members and patients. Family carers were given practical training by clinical research assistants to assess the following vital signs: heart rate, respiratory rate, level of consciousness (using the AVPU scale^16^) and pulse oxygen saturation (SpO_2_). This included ensuring that carers knew how to wash their hands before and after contact with their patients to reduce the risk of infection transmission. Family members were taught to record vital signs on a basic observation chart every 4 h after surgery (Supplementary file). They were taught how to interpret these results with the aid of colour posters displayed throughout each ward (Supplementary file). For simplicity, a single pragmatic cut-off for vital signs was used, allowing family members to decide whether the result they achieved was acceptable or needed attention from a medical worker. This approach has been used successfully by healthcare workers in an intensive care setting.^17^ Family members had access to a simple pulse oximeter that was shared between all patients in their room of the ward during the trial. Training was conducted by the research team one-on-one or in small groups if multiple participants were recruited at the same time either before or immediately after their patient's surgical procedure. The intervention period lasted from arrival on the postoperative ward after surgery until the end of the third postoperative day, although family members were allowed to continue monitoring after this period if they wished. Each day started at 08:00 and ended at 08:00 the following day. The day of surgery was counted as day 0. Patients were followed up daily until discharge from hospital, and if needed, family members received refresher training during these follow-up visits.

### Outcome measures

The primary outcome measure was the frequency of a completed set of vital signs performed per patient during the intervention period. A patient who arrived on the postoperative ward early on day 0 and who was monitored at the standard frequency (six sets of vital signs documented every 24-h period) would have a total of 24 sets of vital signs during the 96-h intervention period. A set of vital signs was defined as three or more of the four vital signs: heart rate, respiratory rate, level of consciousness, and oxygen saturation. Secondary outcomes were all-cause in-hospital mortality, censored at 30 days after surgery, and length of hospital stay.

### Statistical analysis

The trial was designed to provide >90% power (two-sided *P*-value <0.05) to detect a change in the rate of vital signs monitoring from one per patient per 24 h to three per patient per 24 h relative to a locally agreed standard of six sets of vital signs documented per patient per 24-h period. Prior audit data had shown the current rates of vital signs monitoring on the surgical wards at Mbale RRH to be as low as 0. Sample size calculations demonstrated that even a very small sample size of >20 would provide adequate statistical power for the primary outcome. A time-bound recruitment period of 6 months was therefore chosen to allow sequential activation of the four clusters, one new cluster per month.

Analyses followed the intention-to-treat principle, including all patients with a recorded outcome, analysed according to the allocated intervention group. Missing data for baseline covariates that were included in the analysis model were accounted for using mean imputation for continuous variables and the missing indicator approach for missing data for categorical variables. Analysis was conducted using STATA 17 (StataCorp, College Station, TX, USA).^18^ Primary outcome analysis used the number of documented sets of vital signs performed per patient per 24 h from arrival on the postoperative ward to the end of the third postoperative day. A repeated measures negative binomial mixed effects model with a random intercept for patient was used. The model was adjusted for the intervention, period, cluster, surgical risk (calculated using the ASOS Risk Score^19^), and number of days after surgery as fixed effects. The treatment effect is presented as an incidence rate ratio (95% confidence interval [CI]).

The secondary outcome of all-cause in-hospital mortality censored at 30 days was analysed using a logistic regression model. The duration of hospital stay was analysed using a Cox proportional hazards model with fixed effects for period and cluster. A hazard ratio (95% CI) from this model measures the relative probability of hospital discharge between treatment groups, with a hazard ratio of >1 indicating a higher probability of hospital discharge after the introduction of the intervention.

## Results

**Fig 1:**
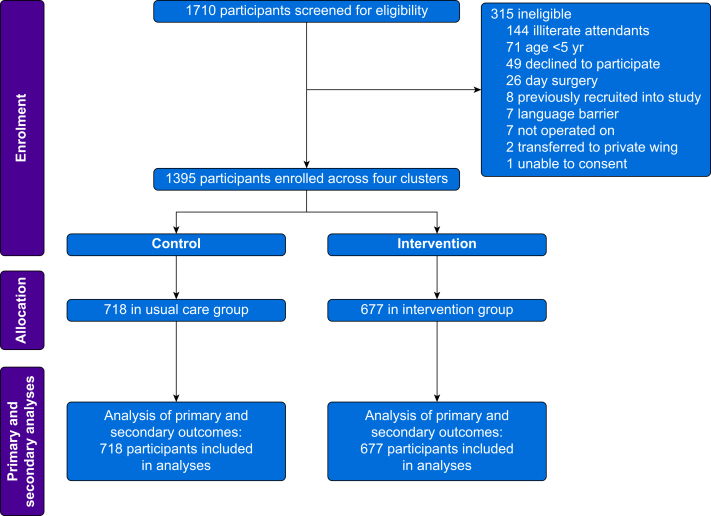
Inclusion of patients in the SMARTER trial.

Between April 13 and October 12, 2021, 1710 participants underwent surgery and were screened for eligibility, with 1395 participants enrolled across four clusters (Fig. 1). The most common reasons for exclusion were literacy and children <5 yr (Fig. 1). There were 718 participants in the usual care group and 677 in the intervention group. The trial was conducted during the second wave of COVID-19 in Uganda. This affected the patient population presenting to Mbale RRH, with the earlier, usual care phase seeing a larger proportion of maternity patients. As a result, control patients were younger and included more women (Table 1 and Supplementary Table S1). There were no patients with missing primary or secondary outcome data.

**Table 1:** Baseline patient characteristics. Data are presented as *n* (%) unless otherwise stated. ASOS, African Surgical Outcomes Study; IQR, interquartile range.

	Overall (*n*=1395)	Usual care (*n*=718)	Intervention (*n*=677)
Age (yr), mean (range)	28.2 (5–89)	26.8 (5–81)	29.7 (5–89)
Sex			
Male	200 (14.3)	55 (7.7)	145 (21.4)
Female	1195 (85.7)	663 (92.3)	532 (78.6)
Comorbid disease			
Hypertension	66 (4.7)	28 (3.9)	38 (5.6)
HIV/AIDS	33 (2.4)	17 (2.4)	16 (2.4)
Diabetes mellitus	27 (1.9)	10 (1.4)	17 (2.5)
ASA physical status			
1	134 (9.6)	49 (6.8)	85 (12.6)
2	1171 (83.9)	629 (87.6)	542 (80.1)
3	81 (5.8)	36 (5.0)	45 (6.6)
4	9 (0.6)	4 (0.6)	5 (0.7)
Surgical procedure category			
Caesarean delivery	1043 (74.8)	610 (85.0)	433 (64.0)
Laparotomy	96 (6.9)	34 (4.7)	62 (9.2)
Orthopaedic	101 (7.2)	19 (2.6)	82 (12.1)
Hernia repair	9 (0.6)	5 (0.7)	4 (0.6)
Gynaecology	20 (1.4)	14 (1.9)	6 (0.9)
Plastics/cutaneous	7 (0.5)	5 (0.7)	2 (0.3)
Ear, nose, and throat	20 (1.4)	4 (0.6)	16 (2.4)
Neurosurgery	16 (1.1)	4 (0.6)	12 (1.8)
Other	83 (5.9)	23 (3.2)	60 (8.9)
Urgency of surgery			
Elective	134 (9.6)	49 (6.8)	85 (12.6)
Urgent	113 (8.1)	24 (3.3)	89 (13.1)
Emergency	1148 (82.3)	645 (89.8)	503 (74.3)
Severity of surgery			
Minor	6 (0.4)	4 (0.6)	2 (0.3)
Intermediate	1182 (84.7)	636 (88.6)	546 (80.7)
Major	207 (14.8)	78 (10.9)	129 (19.1)
Primary indication for surgery			
Infection	65 (4.7)	26 (3.6)	39 (5.8)
Non-communicable disease	168 (12.0)	56 (7.8)	112 (16.5)
Trauma	113 (8.1)	24 (3.3)	89 (13.1)
Caesarean delivery	1049 (75.2)	612 (85.2)	437 (64.5)
ASOS Risk Score, median (IQR)	5 (5–6)	5 (5–6)	5 (5–8)

**Fig 2:**
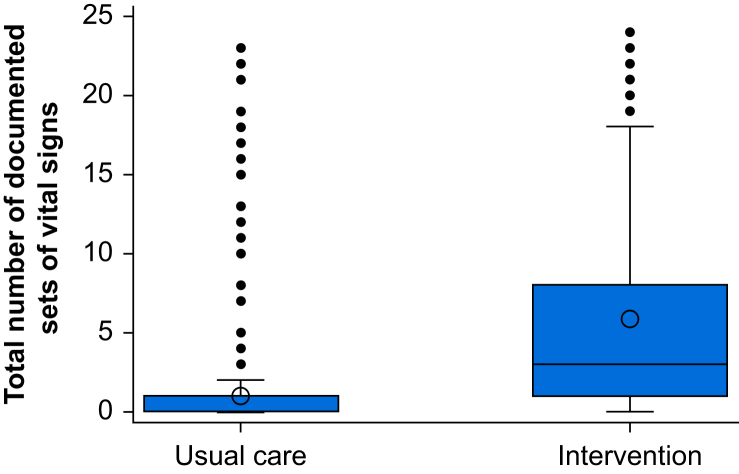
Number of documented vital signs in the usual care and intervention groups.

The median (IQR) number of sets of vital signs monitoring per 3-day study period in the usual care group was 0 (0–1) compared with 3 (1–8) in the intervention group (incident rate ratio 12.4, 95% CI 8.8–17.5, *P*<0.001) (Fig. 2 and Table 2). Mortality at 30 days was 6/718 (0.84%) patients in the usual care group *vs* 12/677 (1.77%) in the intervention group (odds ratio 1.32, 95% CI 0.1–14.7, *P*=0.821) (Table 2). There was no difference between groups in length of hospital stay (usual care: 2 [2–3] days *vs* intervention: 2 [2–4] days; hazard ratio 1.11, 95% CI 0.84–1.47, *P*=0.44) (Table 2).

**Table 2:** Patient outcomes. Data are presented as *n* (%), mean (sd), or median (IQR) unless otherwise stated. ∗Incidence rate ratio. ^†^Odds ratio. ^‡^Hazard ratio. CI, confidence interval.

	Patients with available data, *n* (%)		Summary measure, vital signs per 24 h		Treatment effect (95% CI)	*P*-value
	Control (*n*=718)	Intervention (*n*=677)	Control	Intervention		
Primary outcome: median number of sets of vital signs	718 (100.0)	677 (100.0)	0 (0–1)	3 (1–8)	12.4∗ (8.8–17.5)	<0.001
Secondary outcome: in-hospital mortality	718 (100.0)	677 (100.0)	6 (0.8)	12 (1.8)	1.32^†^ (0.1–14.7)	0.821
Secondary outcome: median duration of hospital stay (days)	718 (100.0)	677 (100.0)	2 (2–3)	2 (2–4)	1.1^‡^ (0.8–1.5)	0.440

## Discussion

The principal finding of this pilot cluster trial is that training family members to assist in vital signs monitoring of patients on hospital wards after surgery is highly feasible, leading to a 12-fold increase in the rate of vital signs measurement. Of the participants screened for eligibility, only 8% (144/1710) were excluded for literacy reasons and 3% (49/1710) were unwilling to participate, suggesting that the intervention was both accepted and not limited by prevailing patient literacy. There was no significant difference in the number of patients dying in-hospital after surgery and no change in the length of hospital stay between the normal care and intervention groups. The findings from this trial add to the growing evidence base that family member involvement in patient care can effectively support the limited numbers of healthcare workers in low-resource settings. Our novel approach to postoperative monitoring is one that warrants further investigation.

Family members are commonly present at the hospital bedside in low-income settings and are an underused resource. Prior work has described the ability of family members to recognise sick patients in the community, especially children with danger signs.^20^, ^21^, ^22^ Despite family members wanting to be empowered to participate more actively, evidence is limited to support their involvement in aspects of inpatient care that are traditionally reserved for healthcare staff.^23^^,^^24^ In some medical specialties such as neonatology, family members are increasingly involved in elements of patient care that have a direct impact on clinical outcomes, for example thermoregulation using kangaroo mother care.^12^^,^^25^ In Uganda, other published examples of direct involvement of family members in inpatient care include the national referral cancer centre, where almost half of family members interviewed reported being involved in providing direct patient care, including giving medication,^26^ and a quality improvement project on the internal medicine wards at the National Referral Hospital in Kampala that was able to double adherence to prescribed inpatient medications by involving family members.^11^

Interventions using family members to help monitor a patient's illness and identify when they are deteriorating are relatively novel. We identified one study from Africa in the general paediatric wards of Kenyatta National Hospital, Kenya, where family member involvement in the recognition of clinical deterioration among acutely ill children in a low-resource setting was a feasible approach to help prioritise professional clinical assessment during hospitalisation.^27^ This study taught caregivers to display colour-coded severity of illness flags based on three perceived signs of clinical deterioration, namely level of consciousness, capillary refill time, and presence of chest retractions (the ‘FASTER’ tool). The FASTER intervention was anticipated to trigger increased frequency of clinician reassessment. Although the study was not powered to detect the impact of the intervention on mortality, 82% of clinicians and 100% of caregivers agreed that the FASTER monitoring tool would improve the care of sick paediatric inpatients in their setting.^13^ Interventions to reduce failure to rescue in high-income countries have been reviewed.^28^^,^^29^ Efforts to reduce failure to rescue need to address the entire sequence of events if they are to be effective.^30^ This starts with improved recognition and includes communication of the deteriorating patient and a team responding appropriately. To see a positive impact on patient outcomes, any intervention increasing recognition of the deteriorating patient through improved vital signs monitoring also needs to ensure the ensuing communication and response is effective.

Strengths of this trial include the iterative development of a pragmatic intervention to improve postoperative monitoring for surgical patients using available resources in a low-income setting and a carefully planned cluster-randomised trial study design. The stepped-wedge approach enabled the intervention to be introduced into an entire ward at the same time and therefore avoided the potential for confusion between intervention and usual care patients who might have been in adjacent beds under the care of the same nurse. Only family members on intervention wards were trained, allowing us to minimise the risk of contamination.

Limitations include those influenced by the COVID-19 pandemic and areas of intervention fidelity we did not test. Uganda was affected by two waves of COVID-19, with the second wave in late April 2021, just after the trial started recruitment. Limited numbers of clinical COVID-19 cases and a strict lockdown across the country allowed us to continue recruitment into the trial during this period. However, it led to reduced access to healthcare services across the country. Priority was commonly given to maternal care, resulting in a higher than expected proportion of females and Caesarean deliveries during the trial. The median total number of documented vital signs in these wards was much lower than in the general surgical and orthopaedic wards, and so it is possible this had an impact on our primary outcome.

Our intervention was intentionally designed to be as simple as possible. Changes in blood pressure are commonly a late sign of clinical deterioration, and blood pressure cuffs left inflated provide an increased risk of harm. For practical reasons, we therefore excluded blood pressure measurement from family member vital signs. Numeracy is an essential requirement for vital sign measurement and recording. We excluded family members who were unable to read numbers from the pulse oximeter (114; 7% of the potential participants screened). We recognise that this choice might have led to higher intervention fidelity; however, our approach was a pragmatic balance between an intervention that 100% of family members could manage and one that included vital signs that have a higher chance of detecting deterioration.^31^ Patients who cannot be supported because of the absence of a family member or absence of a numerate family member might still benefit from being treated in a hospital ward where the workload on nursing staff is offset by our trial intervention. For this reason, a larger effectiveness trial would need to be as inclusive as possible. Such a future trial would also need to consider a longer period of postoperative monitoring to capture complications that occur after 72 h, would need to explore the accuracy of the individual vital signs taken by family members, and would need to be designed to determine the impact of our intervention on patient mortality outcomes.

In conclusion, in the SMARTER pilot trial, family member supplemented monitoring for patients after surgery resulted in a rate of vital signs monitoring that was 12 times greater than in usual care. Our concurrent process evaluation reported elsewhere^14^ provides important lessons that allow interpretation of our trial findings in context. Failure-to-rescue patients who develop complications after surgery is common in low- and middle-income countries, and innovative solutions to address this are needed in the short term to help reduce preventable deaths after surgery. We show that family members can be involved in patient care to supplement the routine monitoring of patients on postoperative wards. This intervention now needs testing to determine its effectiveness on a larger scale.

## Authors’ contributions

Study conception: AHS, FB, TS, RP

Study design: AHS, FB, TS, AP, RP

Data analysis: AHS, AP, RP

Writing the first draft of the manuscript: AHS

Patient recruitment: JuN, JoN, JK, HAM, LN, MK, WW, CM, MS, BE

Data collection: JuN, JoN, JK, HAM, LN, MK, WW, CM, MS, BE

Reviewed, revised, and approved the final manuscript: all authors

## Acknowledgements

The authors would like to extend their thanks to all the patients, family members, and staff at Mbale Regional Referral Hospital who were involved in this pilot study.

## Declaration of interest

RP has received research grants and/or honoraria from Edwards Lifesciences and Intersurgical UK and is an editor of the *British Journal of Anaesthesia*. All other authors have no interests to declare.

## Funding

The UK National Institute for Academic Anaesthesia (RCoA/BJA Project Grant) and the 10.13039/501100000272National Institute for Health Research (NIHR Global Health Group on Perioperative and Critical Care—NIHR133850). The funder had no role in study design, data collection, data analysis, data interpretation, or writing of the report. The trial was sponsored by Queen Mary University of London.

## Data sharing

Requests for data sharing are welcome from bona fide researchers.
