# Corrigendum to ‘Family supplemented patient monitoring after surgery (SMARTER): a pilot stepped-wedge cluster-randomised trial’ (*Br J Anaesth* 2024; 133: 846–52)

**DOI:** 10.1016/j.bja.2025.01.026

**Published:** 2025-02-20

**Authors:** Adam Hewitt-Smith, Fred Bulamba, Akshaykumar Patel, Juliana Nanimambi, Lucy R. Adong, Bernard Emacu, Mary Kabaleta, Justine Khanyalano, Ayub H. Maiga, Charles Mugume, Joanitah Nakibuule, Loretta Nandyose, Martin Sejja, Winfred Weere, Timothy Stephens, Rupert M. Pearse

**Affiliations:** 1 Faculty of Medicine and Dentistry, Queen Mary University of London, London, UK; 2 Department of Anaesthesia and Critical Care, Faculty of Health Sciences, Busitema University, Mbale, Uganda; 3 Elgon Centre for Health Research and Innovation (ELCHRI), Mbale, Uganda; 4 Comprehensive Rehabilitation Services in Uganda (CoRSU) Hospital, Kisubi, Uganda; 5 Nexus Centre for Research and Innovations (NCRI), Wakiso, Uganda

The authors regret that the Funding section of the above article was incorrect. Please see the correct funding information below.

The authors would like to apologise for any inconvenience caused.

## Funding

This research was funded by the UK National Institute for Academic Anaesthesia (RCoA/BJA Project Grant) and the NIHR (NIHR Global Health Group on Perioperative and Critical Care—NIHR133850) using UK international development funding from the UK government to support global health research. The views expressed in this publication are those of the author(s) and not necessarily those of the NIHR or the UK government.

