# Supplementary material for: Family supplemented patient monitoring after surgery (SMARTER): a pilot stepped-wedge cluster-randomised trial
Source: Br J Anaesth. 2024 Jul 27;133(4):846–52. doi: 10.1016/j.bja.2024.06.027 (PMC11443126; doi:10.1016/j.bja.2024.06.027)
Supplement: Multimedia component 1 [file mmc1.docx]

**Supplementary file**

Supplementary Figure 1. Intervention implementation timeline for clusters 1-4

| = baseline = intervention period | | | | | | |
| --- | --- | --- | --- | --- | --- | --- |
| **Cluster 1** |  |  | | | | |
| **Cluster 2** |  | |  | | | |
| **Cluster 3** |  | | |  | | |
| **Cluster 4** |  | | | |  | |
|  | 1 | 2 | 3 | 4 | 5 | 6 |
| Time (months) | | | | | | |

Supplementary Figure 2 - Family member vital signs chart


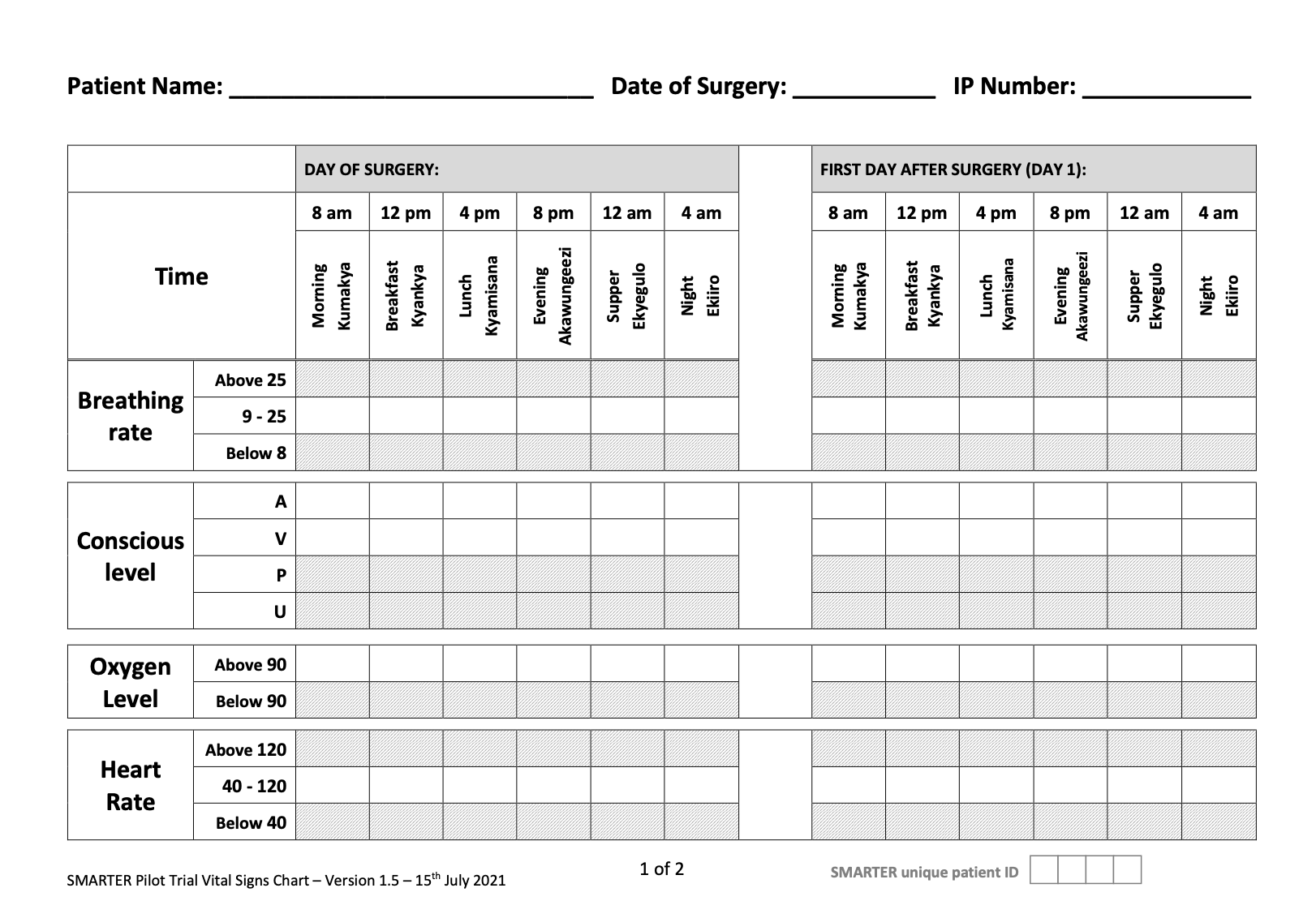


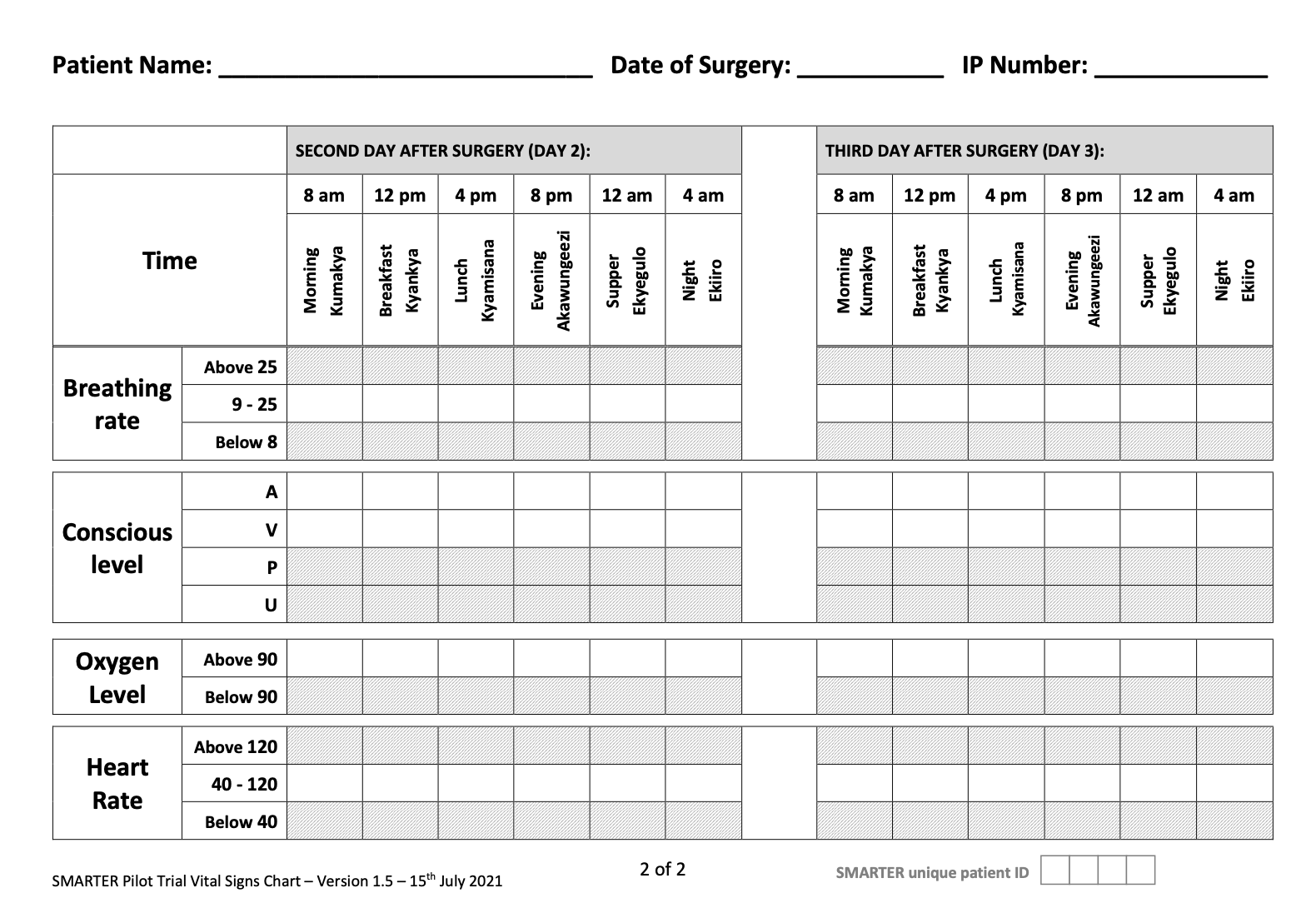


Supplementary Figure 3 - Infographic colour poster

Supplementary Table 1 – Demographics by cluster

|  | **Cluster 1** | | **Cluster 2** | | **Cluster 3** | | **Cluster 4** | |
| --- | --- | --- | --- | --- | --- | --- | --- | --- |
| **Item** | Control  N = 15 | Intervention  N = 95 | Control  N = 71 | Intervention  N = 134 | Control  N = 595 | Intervention  N = 426 | Control  N = 37 | Intervention  N = 22 |
| **Age, mean (SD)** | 31.7 (20.3) | 31.0 (21.3) | 41.1 (20.1) | 42.4 (20.2) | 24.8 (6.2) | 25.6 (6.9) | 29.8 (11.5) | 26.7 (6.9) |
| **Gender**   - Male - Female | 9 (60.0)  6 (40.0) | 62 (65.3)  33 (34.7) | 46 (64.8)  25 (35.2) | 83 (61.9)  51 (38.1) | 0 (0.0)  595 (100.0) | 0 (0.0)  426 (100.0) | 0 (0.0)  37 (100.0) | 0 (0.0)  22 (100.0) |
| **Co-morbid disease:**   - Hypertension; - HIV/AIDS; - Diabetes mellitus; | 0 (0.0)  0 (0.0)  0 (0.0) | 8 (8.4)  2 (2.1)  1 (1.1) | 4 (5.6)  3 (4.2)  6 (8.5) | 17 (12.7)  4 (3.0)  10 (7.5) | 21 (3.5)  14 (2.4)  4 (0.7) | 13 (3.1)  10 (2.3)  6 (1.4) | 3 (8.1)  0 (0.0)  0 (0.0) | 0 (0.0)  0 (0.0)  0 (0.0) |
| **ASA grade**   - I - II - III - IV | 8 (53.3)  6 (40.0)  1 (6.7)  0 (0.0) | 50 (52.6)  40 (42.1)  5 (5.3)  0 (0.0) | 30 (42.3)  25 (35.2)  13 (18.3)  3 (4.2) | 26 (19.4)  66 (49.3)  37 (27.6)  5 (3.7) | 10 (1.7)  568 (95.5)  16 (2.7)  1 (0.2) | 8 (1.9)  415 (97.4)  3 (0.7)  0 (0.0) | 1 (2.7)  30 (81.1)  6 (16.2)  0 (0.0) | 1 (4.5)  21 (95.5)  0 (0.0)  0 (0.0) |
| **Surgical procedure category**   - Caesarean Section - Laparotomy - Orthopaedic - Hernia Repair - Gynaecology - Plastics/cutaneous - Ear, Nose & Throat - Neurosurgery - Other | 0 (0.0)  0 (0.0)  13 (86.7)  0 (0.0)  0 (0.0)  0 (0.0)  2 (13.3)  0 (0.0)  0 (0.0) | 0 (0.0)  1 (1.1)  77 (81.1)  0 (0.0)  0 (0.0)  0 (0.0)  14 (14.7)  0 (0.0)  3 (3.2) | 0 (0.0)  26 (36.6)  6 (8.5)  5 (7.0)  0 (0.0)  5 (7.0)  2 (2.8)  4 (5.6)  23 (32.4) | 0 (0.0)  56 (41.8)  5 (3.7)  2 (1.5)  0 (0.0)  2 (1.5)  2 (1.5)  12 (9.0)  55 (41.0) | 594 (99.8)  1 (0.2)  0 (0.0)  0 (0.0)  0 (0.0)  0 (0.0)  0 (0.0)  0 (0.0)  0 (0.0) | 420 (98.6)  1 (0.2)  0 (0.0)  2 (0.5)  1 (0.2)  0 (0.0)  0 (0.0)  0 (0.0)  2 (0.5) | 16 (43.2)  7 (18.9)  0 (0.0)  0 (0.0)  14 (37.8)  0 (0.0)  0 (0.0)  0 (0.0)  0 (0.0) | 13 (59.1)  4 (18.2)  0 (0.0)  0 (0.0)  5 (22.7)  0 (0.0)  0 (0.0)  0 (0.0)  0 (0.0) |
| **Urgency of surgery**   - Elective - Urgent - Emergency | 7 (46.7)  4 (26.7)  4 (26.7) | 31 (32.6)  53 (55.8)  11 (11.6) | 31 (43.7)  12 (16.9)  28 (39.4) | 34 (25.4)  34 (25.4)  66 (49.3) | 6 (1.0)  2 (0.3)  587 (98.7) | 17 (4.0)  1 (0.2)  408 (95.8) | 5 (13.6)  6 (16.2)  26 (70.3) | 3 (13.6)  1 (4.5)  18 (81.8) |
| **Severity of surgery**   - Minor - Intermediate - Major | 1 (6.7)  8 (53.3)  6 (40.0) | 0 (0.0)  40 (42.1)  55 (57.9) | 3 (4.2)  23 (32.4)  45 (63.4) | 2 (1.5)  59 (44.0)  73 (54.5) | 0 (0.0)  583 (98.0)  12 (2.0) | 0 (0.0)  426 (100.0)  0 (0.0) | 0 (0.0)  22 (59.5)  15 (40.5) | 0 (0.0)  21 (95.5)  1 (4.5) |
| **Primary indication for surgery**   - Infection - Non-communicable disease - Trauma - Caesarean Section | 6 (40.0)  2 (13.3)  7 (46.7)  0 (0.0) | 8 (8.4)  17 (17.9)  70 (73.7)  0 (0.0) | 17 (23.9)  37 (52.1)  17 (23.9)  0 (0.0) | 30 (22.4)  82 (61.2)  19 (14.2)  3 (2.2) | 0 (0.0)  0 (0.0)  0 (0.0)  595 (100.0) | 0 (0.0)  5 (1.2)  0 (0.0)  421 (98.8) | 3 (8.1)  17 (45.9)  0 (0.0)  17 (45.9) | 1 (4.5)  8 (36.4)  0 (0.0)  13 (59.1) |
| **ASOS Risk Score, median (IQR)** | 8.0  (6.5 - 9.0) | 8.0  (6.0 - 11.0) | 10.0  (7.0 - 14.0 | 12  (7.0 - 14.0) | 5.0  (5.0 - 6.0) | 5.0  (5.0 - 6.0) | 7.0  (5.0 - 11.0) | 6.0  (5.0 - 7.0) |
